# Implantable cardioverter defibrillator and cardiac resynchronization treatment in people with type 2 diabetes: a comparison with age- and sex matched controls from the general population

**DOI:** 10.1186/s12933-023-02084-z

**Published:** 2024-01-06

**Authors:** Elina Rautio, Fredrik Gadler, Soffia Gudbjörnsdottir, Stefan Franzén, Lars Rydén, Gianluigi Savarese, Ann-Marie Svensson, Linda G. Mellbin

**Affiliations:** 1 Cardiology Research Unit, Department of Medicine, Solna Karolinska Institutet, 171 76 Stockholm, Sweden; 2 Department of Medicine, Karolinska University Hospital, Huddinge, Stockholm, Sweden; 3 Department of Molecular and Clinical Medicine, University of Gothenburg, Gothenburg, Sweden; 4 Centre of Registers in Region Västra Götaland, Gothenburg, Sweden; 5 Health Metrics Unit, the Sahlgrenska Academy, University of Gothenburg, Gothenburg, Sweden; 6 Heart, Vascular and Neuro Theme, Karolinska University Hospital, Stockholm, Sweden

**Keywords:** Type 2 diabetes, Tachycardia, Implantable cardioverter defibrillator, Registry, Population based

## Abstract

**Background:**

Increased risk of severe tachyarrhythmias is reported in patients with type 2 diabetes mellitus (T2DM). The aim of this study was to explore if treatment with cardiac implantable electronic device (CIED) such as implantable cardioverter defibrillator (ICD), cardiac resynchronization therapy- pacemaker and -defibrillator (CRT-P/CRT-D) differed in patients with vs. without T2DM. A secondary aim was to identify patient characteristics indicating an increased CIED treatment.

**Method:**

416 162 adult patients with T2DM from the Swedish National Diabetes Registry and 2 081 087 controls from the Swedish population, matched for age, sex and living area, were included between 1/1/1998 and 31/12/2012 and followed until 31/12/2013. They were compared regarding prevalence of ventricular tachycardia (VT) at baseline and the risk of receiving a CIED during follow-up. Multivariable Cox regression analysis was performed to estimate the risk of CIED-treatment and factors identifying patients with such risk.

**Results:**

Ventricular fibrillation (VF) (0.1% vs 0.0004%) and (VT) (0.2% vs. 0.1%) were more frequent among patients with T2DM compared to controls. CIED-treatment was significantly increased in patients with T2DM both in unadjusted and adjusted analyses. HR and 95% CI, after adjustment for sex, age, marital status, income, education, country of birth, coronary artery disease and congestive heart failure, were 1.32 [1.21–1.45] for ICD, 1.74 [1.55–1.95] for CRT-P and 1.69 [1.43–1.99] for CRT-D. Blood-pressure and lipid lowering therapies were independent risk factors associated to receiving CIED, while female sex was protective.

**Conclusions:**

Although the proportion of VT/VF was low, patients with T2DM had a higher prevalence of these conditions and increased risk for treatment with CIED compared to controls. This underlines the importance of recognizing that T2DM patients have an increased need of CIED.

**Supplementary Information:**

The online version contains supplementary material available at 10.1186/s12933-023-02084-z.

## Background

In a recent observational report based on the Swedish National Diabetes Registry we showed that the incidence of bradyarrhythmia and pacemaker (PM) treatment was higher in patients with type 2 diabetes mellitus (T2DM) than in an age and sex matched control group without diabetes mellitus (DM) [1]. There are also indications that tachyarrhythmias are more frequently occurring in individuals with DM. Both supraventricular tachyarrhythmias, in particular atrial fibrillation (AF), and ventricular tachyarrhythmias (VT) causing sudden cardiac death (SCD) presumably due to ventricular fibrillation (VF) are reported to be increased in patients with DM [2–5]. DM seems furthermore to be a strong predictor of all-cause mortality in patients presenting with VT [5]. However, studies on the relationship between DM, VT and VF are sparse and with a majority conducted in the context of hypoglycemic episodes [6–8].

Since severe tachyarrhythmias may cause serious symptoms such as syncope and SCD if not immediately treated with cardiopulmonary resuscitation and defibrillation, it is of importance to explore whether VTs are more frequent in patients with DM. Suffering from an out of hospital cardiac arrest has been associated with lower survival odds in patients with vs without DM (OR 0.78, 95% CI 0.68–0.89) and with poorer neurological outcome if surviving and having DM [9]. In a retrospective study of implantable cardioverter defibrillator (ICD) recipients, 28% of patients receiving a primary preventive ICD had DM while the corresponding proportion among those with secondary prevention was 12% [10]. These estimates can be compared with the total global prevalence of DM which was estimated to 9.3% in 2019 [11]. In a study of post-myocardial infarction patients from Finland and Germany, the incidence of SCD was elevated in patients with T2DM compared to patients without DM. The incidence was substantially increased among patients with DM with an ejection fraction < 35% [12].

If there is an increased risk for tachyarrhythmias per se in patients with DM or if it is related to the presence of congestive heart failure (CHF) and/or coronary artery disease (CAD), known common comorbidities in patients with DM, is still an open question. However, data on whether individuals with T2DM receive cardiac resynchronization therapy (CRT) and ICD treatment more frequently than people without T2DM is limited as is information on the indication for such treatment in recipients with T2DM compared to those without T2DM.

The aim of the present study was to explore if treatment with ICD, cardiac resynchronization therapy-pacemaker (CRT-P) and cardiac resynchronization therapy-defibrillator (CRT-D) is increased in patients with T2DM compared to age- and sex matched subjects from the general population without DM. A secondary aim was to identify patient characteristics indicating an increased need for such devices in patients with T2DM.

## Method

### Study cohort

This population-matched cohort study is based on five different national Swedish registries which are described below. The patient cohort (n = 416 162) consists of individuals registered in the Swedish National Diabetes Registry between the dates 1/1 1998 and 31/12 2012 with a T2DM diagnosis and without a previous ICD/CRT-P/D-implantation. Entry point for the study was at the first time of registration in the National Diabetes Registry [13]. The patients were followed until 31/12 2013 or time of death. Date of death during follow up was obtained from the Swedish Cause of Death Registry. For each registered patient with T2DM, five controls (n = 2 081 087) matched for age, sex and living area without previous ICD/CRT-P/D or any DM diagnosis defined as not being registered in the National Diabetes Registry, were randomly selected from the Swedish population registry. The primary endpoint was a de novo ICD/CRT-P/D-implantation. Information on ICD/CRT-P/D treatment including date of implantation and type of device was obtained from the national patient registry applying the diagnostic codes: FPG30, FPG36, FPG33 and FPE26 [14]. Baseline data for previous medical history was obtained from the national patient registry using International Classification of Diseases (ICD) 9 and 10 codes.

### Data sources

The present study is based on data from the following five registries:

*The Swedish National Diabetes Registry*: This registry, which was initiated in 1996, provides nationwide information on patients with DM comprising a majority of individuals who are resident in Sweden with DM aged ≥ 18 years (coverage in 2020 = 87%). Information about clinical characteristics, risk factors, diabetes related complications, and treatments is registered annually or more often in case of change of medication. Data is collected by trained nurses and physicians and include information obtained in primary care and at hospital outpatient clinics [15].

*The longitudinal integration database for health insurance and labour market studies-registry (LISA);* Information on educational level, marital status and country of birth were retrieved from the LISA-registry which is updated annually with information on all who are living in Sweden combining data from the labour market and educational and social sectors.

*The Swedish population registry:* The Swedish population registry includes all Swedish residents since 1968 and comprises information on year and date of birth and sex. To be eligible the control subjects had to be free from any registration of DM in the National Diabetes Registry through the complete study period.

*The National Patient Registry;* Information on previous medical history was obtained from the national patient registry*.* International Classification of Disease (ICD) codes versions 10 and 9 were used for atrial fibrillation, acute myocardial infarction (AMI), CAD, stroke, CHF, end stage renal disease, Atrio-ventricular block (AV-block) I-III, sick sinus syndrome, VT, VF (Additional file 1: Table S1). Patients and controls were defined as having any of the above-mentioned conditions if the ICD code was registered in the patient registry any time before the entry point. The National Patient registry has national coverage from 1987.

*The Swedish Cause of death registry*; Comprises data on all deaths of people registered in Sweden with underlying cause of death, based on ICD codes.

The three latter registries are handled by the Swedish National board of Health and Welfare. All the five registries have been merged to a single dataset linked through personal identification numbers that all Swedish citizens have. After the merging it is anonymized, each subject receiving a personal serial number.

### Definitions

Variables from the *Swedish National Diabetes Registry*:

*Type 2 diabetes mellitus* was defined according to epidemiological criteria as persons treated with diet with or without oral glucose lowering agents or a prescription of insulin with or without concomitant oral glucose lowering agents; the latter category applied only to patients ≥ 40 years at the time for the T2DM-diagnosis [13, 16].

*Glycated hemoglobin c (HbA1c)* was expressed both in mmol/mol and % according to the International Federation of Clinical Chemistry and Laboratory Medicine and Diabetes Control and Complications Trial (DCCT)*.*

*Microalbuminuria* was defined as at least two positive results obtained within 1 year and defined as albumin to creatinine ratio of 3–30 mg/mmol (30–300 mg/g) or urinary albumin clearance of 20–200 µg/min (20–300 mg/L).

*Macroalbuminuria* was defined as an albumin-to-creatinine ratio > 30 mg/mmol (close to 300 mg/g or more) or urinary albumin clearance > 200 µg/min (> 300 mg/L). eGFR was estimated from the creatinine value and calculated using the Chronic Kidney Disease (CKD) Epidemiology Collaboration (CKD-EPI) equation [17].

*End stage renal disease* was defined as the need for renal dialysis, renal transplantation, or an estimated Glomerular Filtration rate (eGFR) of less than 15 mL/min.

*A smoker* was defined as a person who smoked one or more cigarettes per day, or a pipe daily, or who had stopped smoking within the past 3 months.

*Body mass index (BMI)* was calculated using data on weight and height, collected by primary care units and hospital outpatient clinic.

*Blood pressure (BP)* was recorded as the mean of two readings (Korotkoff phases 1–5) with the patient sitting or lying down, using a cuff of appropriate size.

*High density lipoprotein (HDL), low density lipoprotein (LDL),* was measured in mmol/l. 

Variables from the *LISA registry.* The *educational level* was categorised as low (< 9 years), intermediate (10–12 years), or high (college/university).

*Marital status*wad defined as single, divorced, married or widowed.

### Statistical analysis

Baseline characteristics for patients are those collected at their first registration in the National Diabetes Registry while baseline data for controls are those recorded in the national patient registry at the same date as for their respective patient. Continuous variables are presented as mean and standard deviations (SD) and categorical data as numbers (n) and percentages (%). The p-values are assessed using t-tests for continuous variables and chi-square tests for discrete variables. ICD/CRT-P/D implantations performed during follow-up are presented as numbers (n) and percentages (%) while the crude incidence of ICD/CRT-P/D implantations during the time of observation is expressed as number of events/100 000 person-years. The risk for a first ICD/CRT-P/D implantation in individuals with or without T2DM is assessed by Cox proportional hazard regression and presented as hazard ratio (HR) and 95% confidence intervals (CI). Adjustments are performed in three models. Model 1: age, sex, marital status, educational level and country of birth (Sweden, Europe, outside Europe). Model 2: as in Model 1 and in addition CAD and Model 3: as in Model 1 and 2 and in addition CHF. The primary endpoint is de novo implantation of ICD/CRT-P/D. Persons who died are censored at the time of death.

The crude cumulative risk of ICD/CRT-P/D implantations is presented through Kaplan–Meier curves and assessed by log-rank test for individuals with and without T2DM.

In order to establish a risk factor profile related to the need for ICD/CRT-P/D we used Cox progression hazard multivariate analysis, with the following baseline characteristics: age, diabetes duration, HbA1c, systolic BP, diastolic BP, BMI, HDL, LDL, eGFR, female sex, micro- and microalbuminuria, lipid and blood pressure lowering drugs and smoking. The HR described are per one unit change for the respective continuous variable, for example, one unit of change in mmol/mol for HbA1c. This analysis was only performed in patients with T2DM (n = 97 826), who had all these variables registered in the National Diabetes Registry.

For all analyses, a two-sided p-value < 0.05 was considered statistically significant. The analyses were performed in R version 4.0.2.

## Results

### Baseline characteristics

In the present study, a total of 416 162 patients with T2DM and 2 081 087 controls were identified and included. The average and median follow up time was 8.0 and 7.3 years respectively. Baseline characteristics of patients and controls are presented in Table 1. The mean age at baseline was 64.1 years and 45.7% of the study cohort were females. Patients with T2DM had a more frequent history of AMI (8.5% vs. 3.8%), CAD (16.1% vs 7.7%) and CHF (5.8% vs. 2.5%) compared to controls. The prevalence of a tachyarrhythmia diagnosis, defined as the presence of AF (6.5% vs. 4.0%), VF (0.1% vs 0.0004%) or VT (0.2% vs. 0.1%) was higher in patients with T2DM.

**Table 1:** Baseline characteristics

Clinical characteristics	Type 2 diabetes mellitus n = 416 162	Controls n = 2 081 087	p-value
Age (years*)*	64.1 (12.3)	64.1 (12.3)	
Female sex	190 278 (45.7)	951 478 (45.7)	
Age at diagnosis	58.5 (12.7)	–	
Duration of diabetes at entry into registry (years)	5.5 (7.0)	–	
BMI (k/m^2^)	30 (5.4)	–	
Medical history			
Smoking	54 581 (16.3)	–	
Systolic blood pressure (mm/Hg)	140.0 (18.3)	–	
Diastolic blood pressure (mm/Hg)	78.9 (9.9)	–	
Acute myocardial infarction	35 386 (8.5)	79 016 (3.8)	< 0.0001
Coronary heart disease	67 130 (16.1)	160 921 (7.7)	< 0.0001
Stroke	25 285 (6.1)	74 276 (3.6) 51 653 (2.5)	< 0.0001
Heart failure	24 134 (5.8)		< 0.0001
Amputation	1 444 (0.3)	1 573 (0.1)	< 0.0001
End stage renal disease	954 (0.2)	2432 (0.1)	< 0.0001
Arrythmias			
Atrial fibrillation	27 207 (6.5)	82 971 (4.0)	< 0.0001
Ventricular tachycardia	979 (0.2)	2951 (0.1)	< 0.0001
Ventricular fibrillation	302 (0.1)	882 (0.0004)	< 0.0001
AV-block I	500 (0.1)	1701 (0.1)	< 0.0001
AV-block II	275 (0.1)	852 (0.04)	< 0.0001
AV-block III	710 (0.2)	2212 (0.1)	< 0.0001
Sick sinus syndrome	1120 (0.3)	4184 (0.2)	< 0.0001
Laboratory findings			
HbA1c (mmol/mol)	54.6 (15.0)	–	
HbA1c (%)	7.1 (3.5)	–	
LDL (mmol/liter)	3.0 (1.0)	–	
HDL (mmol/liter)	1.3 (0.4)	–	
Triglycerides	1.9 (1.2)	–	
Micro albuminuria	35 405 (15.1)	–	
Macro albuminuria	19 277 (6.63)	–	
Estimated GFR (ml/min/1.73 m^2^)	81.7 (25.2)	–	
Treatments			
Statins	155 410 (39.9)	–	
Antihypertensive medication	246 912 (63.2)	–	
Diabetes treatment			
Diet only	156 368 (37.6)	–	
Oral drugs	178 642 (42.9)	–	
Insulin	42 631 (10.3)	–	
Oral drugs and insulin	38 521 (9.3)	–	
Marital status			
Married	222 432 (53.4)	1 166 127 (56)	
Separated	70 218 (16.9)	334 839 (16.1)	
Single	67 236 (16.2)	321 102 (15.4)	
Widowed	56 276 (13.5)	258 923 (12.4)	
Educational level (years)			
≥ 9	174 081 (42.8)	727 966 (35.6)	
10–12	166 527 (40.9)	816 933 (39.9)	
College/university	66 191 (16.3)	502 403 (24.5)	
Country of birth			
Sweden	339 403 (81.6)	1 818 840 (87.4)	
Europe except Sweden	44 941 (10.8)	189 765 (9.1)	
Rest of the world	31 818 (7.6)	72 482 (3.5)	

Data from the first inclusion day in the NDR for patients with type 2 diabetes mellitus and the same date for controls. Categorical variables are presented as n (%) and continuous variables as mean (SD)

### ICD and CRT-P/D treatment

**Fig. 1:**
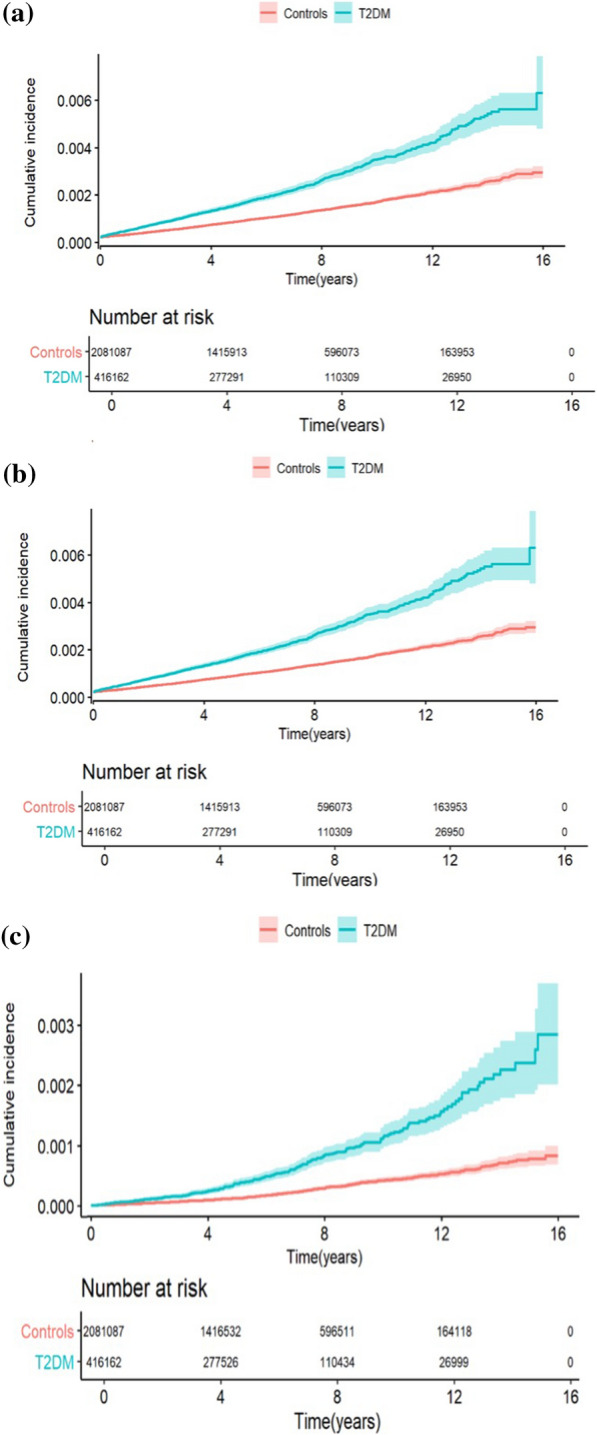
**a** The cumulative risk (including 95% CI) of receiving an ICD in patients with type 2 diabetes mellitus compared to controls. Numbers below the figure represent individuals at risk. **b**. The cumulative risk (including 95% CI) of receiving a CRT-P in patients with type 2 diabetes mellitus compared to controls. Numbers below the figure represent individuals at risk. **c**. The cumulative risk (including 95% CI) of receiving a CRT-D in patients with type 2 diabetes mellitus compared to controls. Numbers below the figure represent individuals at risk

The crude incidence rate for ICD/CRT-P/D was significantly higher in patients with T2DM than in controls, Table 2. The incidence per 100 000 person years for ICD implantation was 30.3 [28.2–32.6] in patients with T2DM vs 14.3 [13.7–14.99] in the control population. For CRT-P the incidence was 19.0 [17.4–20.8] vs 7.4 [6.9–7.8] and for CRT-D; 9.8 [8.6–11.2] vs. 3.5 [3.2–3.8]. The difference increased over time as outlined in the Kaplan Meier curves, Figures 1a-c.

**Table 2:** Incidence/100. 000 person years with 95% CI by type of ICD, CRT-P and CRT-D

	Type 2 diabetes mellitus	Controls
ICD	30.3 [28.2–32.6]	14.3 [13.7–14.99]
CRT-P	19.0 [17.4–20.8]	7.4 [6.9–7.8]
CRT-D	9.8 [8.6–11.2]	3.5 [3.2–3.8]

*CRT-D* cardiac resynchronization therapy- defibrillator, *CRT-P* cardiac resynchronization therapy-pacemaker, *ICD* implantable cardioverter defibrillator

**Fig. 2:**
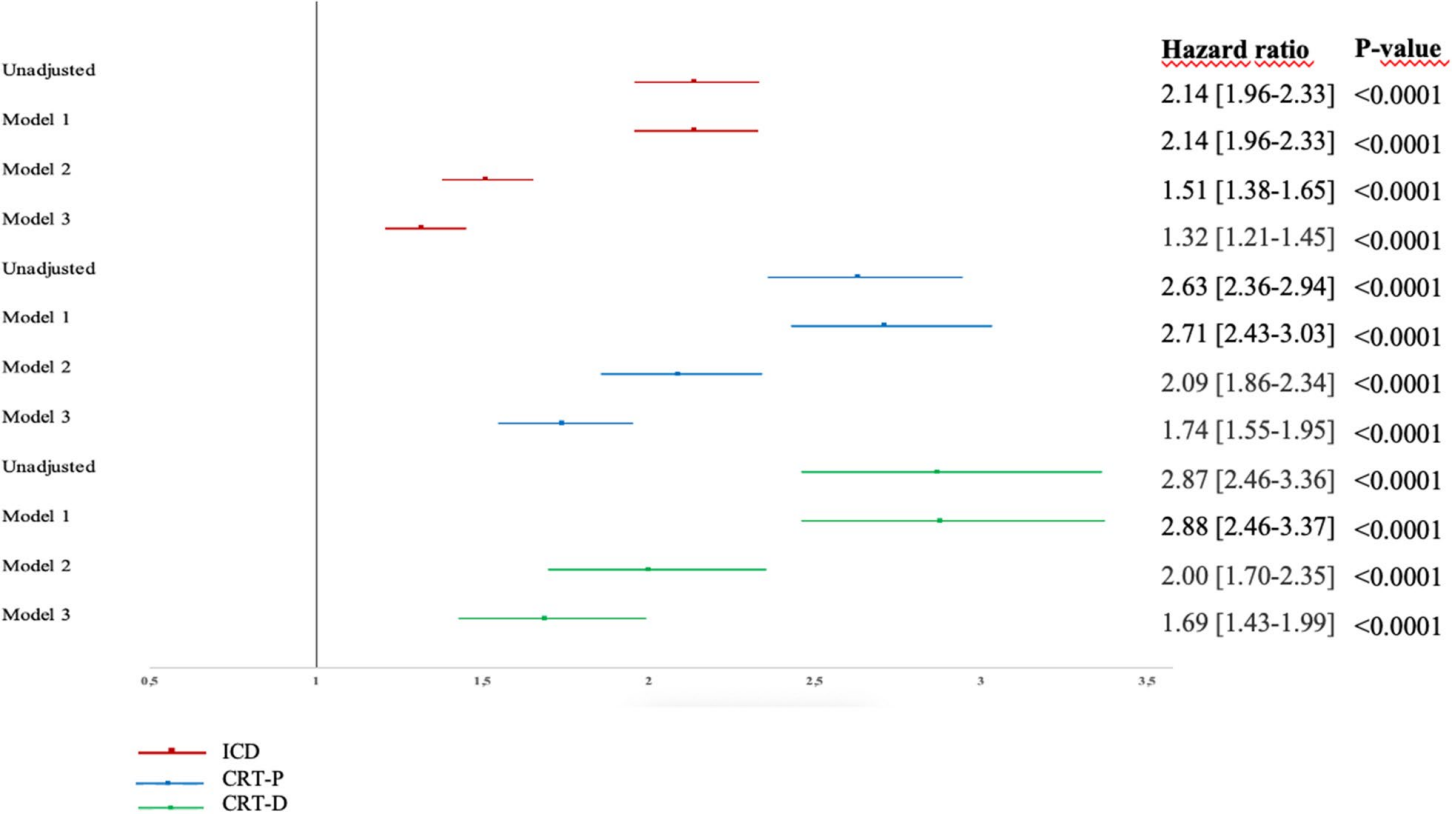
The risk of receiving ICD, CRT-P and CRT-D in patients with type 2 diabetes mellitus compared to controls

The risk of receiving ICD, CRT-P or CRT-D during follow-up was significantly higher in patients with T2DM as shown in Table 3. This was seen in unadjusted Cox regression hazard analyses and remained after adjustments for potential confounders in the three models. The risk decreased when CAD and CHF were included in the analyses but remained significant, Figure 2.

**Table 3:** The risk of receiving ICD, CRT-P and CRT-D in patients with type 2 diabetes mellitus compared to controls

	Hazard ratio [95% CI]	P-value
ICD		
Unadjusted	2.14 [1.96–2.33]	< 0.0001
Model 1^a^	2.14 [1.96–3.33]	< 0.0001
Model 2^b^	1.51 [1.38–1.65]	< 0.0001
Model 3^c^	1.32 [1.21–1.45]	< 0.0001
CRT-P		
Unadjusted	2.63 [2.36–2.94]	< 0.0001
Model 1^a^	2.71 [2.43–3.03]	< 0.0001
Model 2^b^	2.09 [1.86–2.34]	< 0.0001
Model 3^c^	1.74 [1.55–1.95]	< 0.0001
CRT-D		
Unadjusted	2.87 [2.46–3.36]	< 0.0001
Model 1^a^	2.88 [2.46–3.37]	< 0.0001
Model 2^b^	2.00 [1.70–2.35]	< 0.0001
Model 3^c^	1.69 [1.43–1.99]	< 0.0001

*CI* confidence interval, *ICD* Implantable cardioverter defibrillator, *CRT-P* cardiac resynchronization therapy-pacemaker, *CRT-D* cardiac resynchronization therapy-defibrillator

^a^Model 1: Adjusted for sex, age, marital status, income, education, country of birth

^b^Model 2: Adjusted for model 1 and in addition previous CAD

^c^Model 3: Adjusted for the model 2 and in addition previous CHF

### Factors predicting ICD and CRT-P/D -treatment in patients with T2DM

**Fig. 3:**
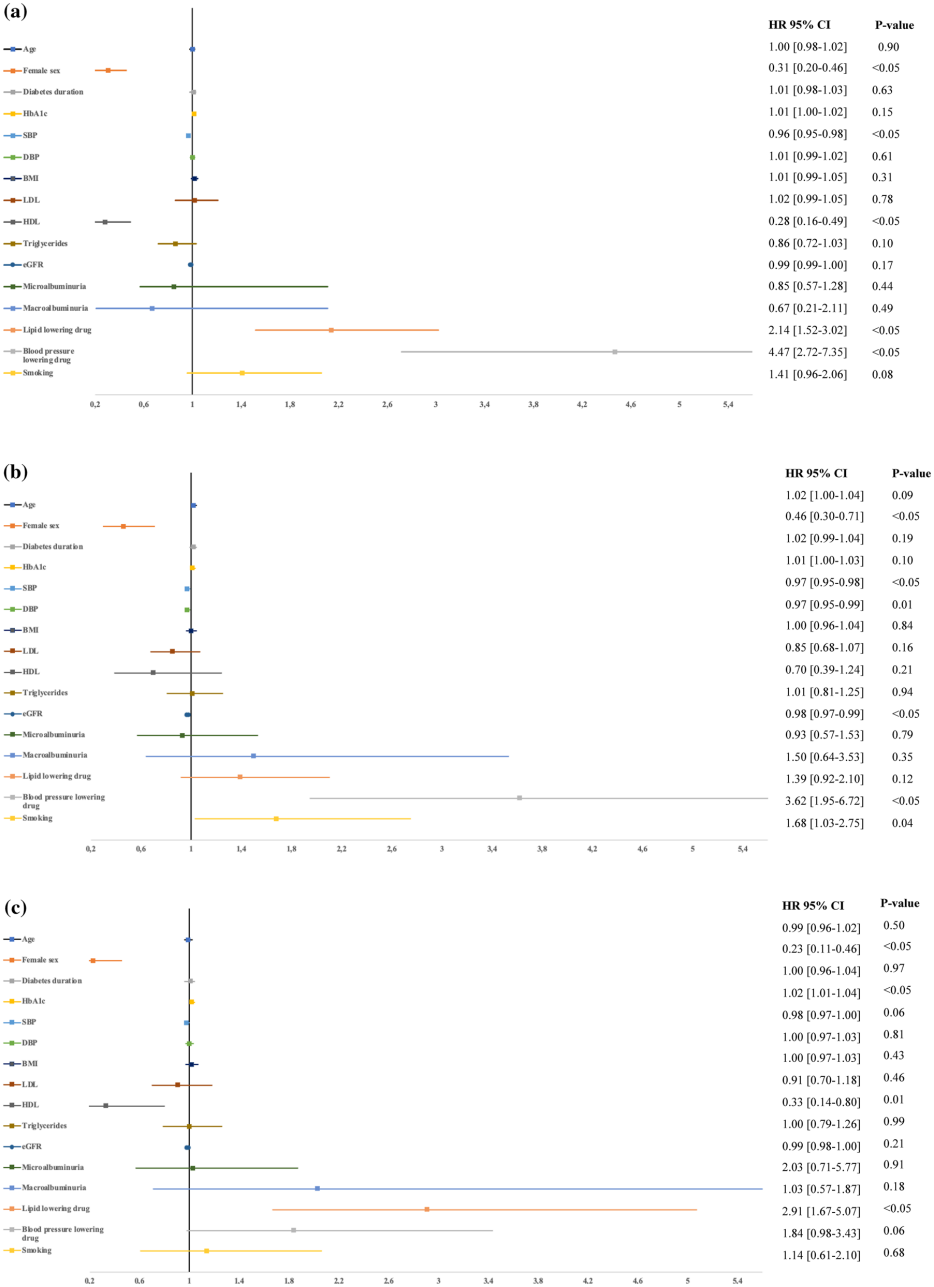
**a** Risk profile of receiving an ICD in patients with type 2 diabetes mellitus presented as HR [95%CI] per one unit change for the variable. **b**. Risk profile of receiving an CRT-P in patients with type 2 diabetes mellitus presented as HR [95% CI] per one unit change for the variable. **c**. Risk profile of receiving an CRT-D in patients with type 2 diabetes mellitus presented as HR [95% CI] per one unit change for the variable. SBP systolic blood pressure; DBP diastolic blood pressure, *BMI* body mass index, *LDL* low density lipoprotein, *HDL* high density lipoprotein, *eGFR* estimated glomerulus filtration rate

Variables to assess the association to receiving an ICD, CRT-P and CRT-D at baseline, was available in a total of 97 826 patients with T2DM and are presented separately in Fig. 3a–c.

As outlined in Fig. 3a, the use of lipid lowering medication (HR 2.14 [1.52–3.02]) and blood pressure lowering medication (HR 4.47 [2.72–7.35]) were significant independent predictors for receiving an ICD. In contrast, female sex (HR 0.31 [0.20–0.46]), higher baseline HDL-cholesterol (HR 0.28 [0.16–0.49]) and higher systolic blood pressure by one unit (HR 0.96 [0.95–0.99]) were associated with a lower likelihood of receiving an ICD.

Patient characteristics associated to receiving a CRT-P were increasing age by one year (HR 1.02 [1.00–1.04]), the use of blood pressure lowering medication (HR 3.62 [1.95–6.72]) and smoking (HR 1.68 [1.03–2.75]) (Fig. 3b). Female sex (HR 0.46 [0.30–0.71]), higher systolic blood pressure (HR 0.97 [0.95–0.98]), higher diastolic blood pressure (HR 0.97 [0.95–0-99]) as well as lower eGFR (HR 0.98 [0.97–0.99]) were associated with lower likelihood of receiving a CRT-P. These findings are reported in Fig. 3b.

Figure 3c depicts that the factors associated to receiving a CRT-D which were higher HbA1c (HR 1.02 [1.01–1.04]) and the use of lipid lowering drugs (HR 2.91 [1.67–5.07]). Female sex (HR 0.23 [0.11–0.46]) and higher HDL-cholesterol (HR 0.33 [0.14–0.80]) were associated with less likelihood of receiving this device.

## Discussion

The main result of this large, population-based cohort study based on real world data was that tachyarrhythmias, although in general uncommon, were significantly more frequent among patients with T2DM than in matched controls. Accordingly, the need for ICD, CRT-P or CRT-D treatment was higher in patients with T2DM compared to controls during follow up. The current results are in line with previous investigations showing that serious ventricular tachyarrhythmias (VT and VF) are more common in individuals with DM compared to those without DM [4, 18, 19]. This study further showed that patients with T2DM have a higher need for CIED and that in particular treatment with blood pressure and lipid-lowering therapies were associated with higher likelihood of receiving any of these devices whereas female sex was associated with less need.

Even though the proportion of patients with T2DM with a history of VT or VF was small (0.2 and 0.1%) it was still significantly higher than in the matched control group. Similar findings were presented by Movahed et al. who reported that 0.2% of patients with DM had a history of VF vs 0.1% in a control group with hypertension but without DM [18]. The low prevalence of VT and VF in the present study and in the report by Movahed et al. may be explained by underreporting of ventricular tachyarrhythmias in some cases due to out of hospital mortality caused by ventricular arrhythmias and the difficulties in capturing such events. The risk for SCD is increased in patients with DM compared to subjects without DM [20–22] suggesting that malignant arrhythmias are increased in this patient population.

The higher prevalence of VT/VF already at baseline in patients with T2DM suggests an increased need for ICD. Both the prevalence and incidence of ICD implantation was indeed increased in patients with T2DM compared to the control population. The low prevalence of VT/VF may be related to underreporting, but it may also be that there are other reasons for ICD treatment. Unfortunately, the present data do not disclose whether the indication for implantations related to primary or secondary prevention, but in 2019 ≅ 60% of all Swedish ICDs were implanted for primary prevention. According to the Swedish pacemaker and ICD registry’s annual report from 2019, CHF was the main indication in 34% of primary preventive ICDs. Other symptoms and indications for ICD implantations, according to the same registry, were syncope in 12.8%, breathlessness/tiredness in 2.9%, palpitations in 4.9%, primary prevention (asymptomatic) in 16.3%, asymptomatic VT/VF in 2.9% and aborted sudden death in 21.6% [23].

A reasonable assumption is that the higher need for ICD therapy in patients with T2DM, at least partly but not only, is explained by the higher prevalence of CHF at baseline. In the present study the prevalence of CHF was twice as common in patients with T2DM compared to controls, 5.8 vs 2.5%. The incidence of ICD/100.000 person years was also twice as high, 3.0 vs 1.4 in patients with T2DM compared to controls. Importantly, it remained higher even after adjusting for other factors including CHF. In patients with DM, the prevalence of CHF ranges between 9 and 22%, which is four times higher than in the general population [24].

Although the present data does not permit a detailed analysis on why patients with DM have a higher risk for arrhythmias needing treatment it is likely that factors related to T2DM per se, among them hypoglycemia and autonomic neuropathy, contribute. CAD is a risk factor for tachyarrhythmias and a subsequent need for ICD [25]. Indeed, the risk for ICD decreased after adjustments for CAD. This supports the findings by Manuchehry et al. showing an overall higher prevalence of DM in patients receiving both primary and secondary preventive ICD [10], but in general there are very few studies describing the need for an ICD in patients with DM and most studies do not differ between type 1 or type 2 diabetes.

Similar to the results for ICD, the incidence for CRT-P and CRT-D implantation was higher in patients with T2DM than in the control population. The elevated risk remained after adjusting for sex, age, marital status, income, education, country of birth, CAD and CHF. Since the indication for CRT, both with and without an adjunct ICD, is CHF, it underlines the important role of CHF as a predictor of the future fate of patients with T2DM. CHF is one of the major cardiovascular complications in patients with T2DM and increases the risk of morbidity and mortality [26, 27] and notable CHF can be partly prevented. The cause of death in patients suffering from CHF is not only a result of hemodynamic failure but also related to ventricular tachyarrhythmias. Interestingly the risk for a CRT-P or CRT-D was attenuated after including CAD in the model highlighting the fact that these different manifestations of cardiovascular disease coincide in patients with T2DM. A variable associated for the need of an ICD or CRT-P was the use of blood pressure lowering drugs that are a marker of hypertension and furthermore often used to treat CHF. A reasonable explanation is that many of them are used for treating CHF, and among them beta-blockers, diuretics and ACE-inhibitors. Lipid-lowering therapy, one of the cornerstones of CAD treatment was also associated with an increased risk for ICD and CRT- D supporting the notion that prevalent CAD is important in this context. Increasing HbA1c had a HR 1.01 [1.00, 1.02] for ICD, HR, 1.01 [1.00, 1.03] for CRT-P and HR 1.02 [1.01, 1.04] for CRT-D, but it did not reach statistical significance for ICD and CRT-P. Finally female sex was associated with a reduced risk for device implantation. The lower risk of a device associated with female sex may have several reasons. Female representation in cardiovascular trials has historically been low [28, 29]. If there are less evidence-based preventive, diagnostic, and therapeutic options for women with CVD, this may result in clinicians being more reluctant to use such therapies in females leading to under treatment and a lower quality of care in comparison with men. In a cross-sectional study by Chatterjee et al. studying patients undergoing CRT implantation in the United States between 2006 and 2012 females were less likely to be referred for CRT implantation than males although the predicted efficacy was greater in women. The authors explained these results as if women had comorbidities decreasing the benefit of CRT treatment decreasing the treatment indication, and that subgroups of males receiving CRT despite an absence of a true benefit of such treatment [30]. These results are in line with a study by Curtis et al. who analyzed patients receiving ICD. Males were 3.2-fold more likely to receive a device for primary prevention and 2.4 times more likely to receive a device for secondary prevention. The authors were unable to identify sex specific risk factors explaining their results [31]. These findings underline the importance of further studies in this field.

DM, being risk factor for heart failure and arrhythmias, and the need of subsequent ICD and CRT therapy has been acknowledged in previous studies and guidelines [12, 32, 33]. In recent guidelines it is suggested to take patients with DM into consideration as a high-risk group and to assess the need for CIED to optimize selection of device therapy and improve outcomes [32]. The current study shows indeed that more patients do receive such the devices, but more studies are needed to further understand if guideline recommendations are implemented or if other factors also contribute. An interesting aspect would be to further investigate if the indications for CIED differs between people with and without DM and if new DM drugs, shown to be cardioprotective, influence the outcome in this context.

### Strengths and limitations

A strength with the present study is the large-scale data with a high number of patients with T2DM representing a well-defined population seen in daily practice and the like-wise large age- and sex-matched control group. Furthermore, the report is based on nationwide high-quality registries with high coverage [14, 15]. Finally, analyses were performed over a long follow-up period. Our study has also several limitations. Patients included in this study were registered in the National Diabetes Registry between the years of 1998–2013 and treatment pattern have changed thereafter. For example, drugs with cardioprotective effects e.g. Sodium-glucose co-transporter-2 (SGLT2) inhibitors and glucagon like peptide (GLP-1) receptor agonists (GLP-1 RAs) have been introduced as a glucose lowering drug. There is a possibility that the use of such drugs may affect the incidence of arrhythmias. Today, guidelines for DM and CVD recommend the use of these drugs due to their cardiovascular benefits [32]. The present study still adds important information regarding the prevalence of arrhythmias in a patient group with T2DM and without treatment with SGLT2 inhibitors and or GLP-1 RAs which is important not the least since many patients with a diagnosis of T2DM are not treated with such drugs. It is of interest to further investigate the impact of these new cardioprotective drugs during a later time frame when the use of these drugs have been more common. In particular since these drugs are less prone to increase hypoglycemia compared to older diabetes medications for example sulphonylureas [34, 35]. Moreover, during the last two decades the guidelines on ICD and CRT-D has changed e.g. in the early years of the present study, i.e. during the 90–00 s the evidence on the beneficial effects of CRT were sparse and hence the recommendations were limited. An important change in both American and European guidelines was the broadening of ICD indications. In the 2002 American guidelines on ICD implantation included both primary and secondary prevention also including patients with heart failure compared to previously only being recommended to patients surviving a life threatening arrythmia. The current ESC guidelines on diabetes and cardiovascular disease emphasize the risk for SCD in patients with DM and CHF and underscore that patients with DM and occurring ventricular arrhythmia or symptoms suggestive of HF should be examined for the presence of an underlying structural heart disease and their eligibility for an ICD should be assessed; this is however a general principle in managing patients with HF, irrespective of diabetes status [32, 36–38].

This is an observational study and therefore there we could not rule out a residual role for known and unknown confounders in our analyses. An important aspect to further investigate is indeed whether, the indications (eg. primary or secondary prophylaxis) for ICD and CRT/-D differ between patients with and without T2DM and also if guideline recommendations are implemented.

## Conclusion

Patients with T2DM receive implantable cardioverter defibrillator, cardiac resynchronization therapy pacemaker or cardiac resynchronization therapy defibrillator more often than people without T2DM. This may at least partly relate to a higher proportion of ventricular tachycardia or ventricular fibrillation, but also to other conditions such as coronary heart disease and in particular congestive heart failure. This risk needs to be taken into consideration when following patients with T2DM. The present study indicates several factors that should be considered in patients with T2DM when assessing the risk for ventricular tachyarrhythmias, including that female sex was associated with less CIED implantations. These findings indicate the importance for the medical community not only to recognize that patients with T2DM are at increased risk for tachyarrhythmias and/or CHF with a subsequent increased need of ICD and CRT-P/D but also to improve treatment of risk factors and implementing guideline recommended therapies. More studies are however needed to understand the relationship between diabetes and arrhythmias.

### Supplementary Information

**Additional file 1: Table S1.** ICD-codes.

## Data Availability

Data are available from the sources stated in the paper on request to the data providers, fulfilling legal and regulatory requirements and with permission from the Swedish Ethical Review Authority of Ministry of Sweden.

## Abbreviations

AF: Atrial fibrillation
AMI: Acute myocardial infarction
AV: Atrio-ventricular
BMI: Body mass index
BP: Blood pressure
CAD: Coronary artery disease
CVD: Cardiovascular disease
CHD: Coronary heart disease
CHF: Congestive heart failure
CI: Confidence interval
CIED: Cardiac implantable electronic device
CKD: Chronic kidney disease
CRT-D: Cardiac resynchronization therapy-defibrillator
CRT-P: Cardiac resynchronization therapy-pacemaker
DBP: Diastolic blood pressure
DM: Diabetes mellitus
eGFR: Estimated glomerulus filtration rate
GLP-1: Glucagon-like peptide 1
LDL: Low density lipoprotein
HbA1c: Glycated hemoglobin A1C
HDL: High density lipoprotein
HR: Hazard ratio
ICD: Implantable cardioverter defibrillator
ICD: International classification of diseases
LISA: Longitudinal integration database for health insurance and labour market studies
MI: Myocardial infarction
PM: Pacemaker
SBP: Systolic blood pressure
SCD: Sudden cardiac death
SD: Standard deviation
SGLT-2: Sodium glucose linked co transporter 2
T2DM: Type 2 diabetes mellitus
VF: Ventricular fibrillation
VT: Ventricular tachycardia
