# Supplementary material for: Implantable cardioverter defibrillator and cardiac resynchronization treatment in people with type 2 diabetes: a comparison with age- and sex matched controls from the general population
Source: Cardiovasc Diabetol. 2024 Jan 6;23:18. doi: 10.1186/s12933-023-02084-z (PMC10771698; doi:10.1186/s12933-023-02084-z)
Supplement: Supplementary file 1 — Additional file 1: Table S1. ICD-codes. [file 12933_2023_2084_MOESM1_ESM.docx]

**Table S1 ICD-codes**

| **ICD 9 and 10 codes** | **Diagnose** |
| --- | --- |
| (I20–I25) | Coronary heart disease (CHD) |
| (I50) | Heart failure (HF) |
| (I61–I64) | Stroke |
| (V42A, V45B, V56A, V56W, Z940, Z491, Z492, Z992*)* | End stage renal disease |
| (NHQ09, NHQ11, NHQ12, NHQ13, NHQ14, NHQ16, NHQ17, NHQ99, NGQ09, NGQ19, NGQ99, NFQ19, NFQ99) | Amputation in the lower extremities NHQ= foot, NGQ= Knee or below, NFQ= above knee |
| I44.0 | AV-block I |
| I44.1 | AV-block II |
| I44.2 | AV-block III |
| I49.5 | Sick sinus syndrome |
| I48.0-2 | Atrial fibrillation |
| I47.2 | Ventricular tachycardia (VT) |
| I49.0 | Ventricular fibrillation (VF) |
| I47.1 | Atrio ventricular nodal reentry tachycardia (AVNRT) |
| FPG30 | Implantation of transvenous cardioverter-defibrillator with generator and ventricular electrode |
| FPG33 | Implantation of transvenous cardioverter-defibrillator and atrial and ventricular electrodes |
| FPG36 | Implantation of transvenous cardioverter-defibrillator with generator and biventricular electrodes |
| FPE26 | Implantation of transvenous pacemaker with biventricular electrodes |
